# Fatiguing freestyle swimming modifies miRNA profiles of circulating extracellular vesicles in athletes

**DOI:** 10.1007/s00421-023-05167-7

**Published:** 2023-05-12

**Authors:** Zhijie Lai, Wentao Lin, Xu Yan, Xiaobin Chen, Guoqin Xu

**Affiliations:** 1 Graduate School, Guangzhou Sport University, Guangzhou, 510500 China; 2 College of Physical Education, Guangzhou College of Commerce, Guangzhou, 511363 China; 3 College of Exercise and Health, Guangzhou Sport University, Guangzhou, 510500 China; 4 Institute for Health and Sport, Victoria University, Melbourne, 3011 Australia; 5 Australia Institute for Musculoskeletal Sciences, Melbourne, VIC Australia; 6 Department of Medicine-Western Health, The University of Melbourne, Melbourne, VIC Australia

**Keywords:** 1500-m freestyle, Extracellular vesicles, miRNA profile, Exercise-induced fatigue, Acute exercise

## Abstract

Extracellular vesicles (EVs) are secreted by various tissues and cells under normal physiological or pathological conditions. Exercise-induced EVs may be involved in the adaptation of exercise-induced fatigue. The 1500-m freestyle is the longest pool-based swimming event in the Olympic Games, and there is a paucity of information regarding changes in the miRNA profiles of circulating EVs after a single session of fatiguing swimming. In this study, 13 male freestyle swimmers conducted a fatiguing 1500-m freestyle swimming session at the speed of their best previously recorded swimming performance. Fasting venous blood was collected before and after the swimming session for analysis. 70 miRNAs from the circulating EVs were found to be differentially expressed after the fatiguing 1500-m freestyle swimming session, among which 45 and 25 miRNAs were up-regulated and down-regulated, respectively. As for the target genes of five miRNAs (miR-144-3p, miR-145-3p, miR-509-5p, miR-891b, and miR-890) with the largest expression-fold variation, their functional enrichment analysis demonstrated that the target genes were involved in the regulation of long-term potentiation (LTP), vascular endothelial growth factor (VEGF), glutathione metabolism pathway, dopaminergic synapse, signal transmission, and other biological processes. In summary, these findings reveal that a single session of fatiguing swimming modifies the miRNAs profiles of the circulating EVs, especially miR-144-3p, miR-145-3p, miR-509-5p, miR-891b, and miR-890, which clarifies new mechanisms for the adaptation to a single session of fatiguing exercise from the perspective of EV-miRNAs.

## Introduction

Extracellular vesicles (EVs), including in particular exosomes and microvesicles, are widely present in various humoral samples, such as blood, urine, saliva, etc. Various tissues and cells in the body have the ability to secrete EVs under normal physiological or pathological conditions. Studies have found that EVs are rich in various communication substances, such as nucleic acids, proteins, mRNA, and microRNAs (miRNAs). Among them, miRNAs play a key role in the exercise-regulated skeletal muscle energy metabolism (Zhang et al. 2018; Whitham et al. 2018; Hou et al. 2019). In recent years, studies have confirmed that acute exercise increases circulating EVs, and exercise intensity influences the response of EVs to endurance exercise (Amosse et al. 2018; Wilhelm et al. 2017). EVs can promote the repair and regeneration of skeletal muscle, enhance the growth of nerve cells, and inhibit the differentiation of neurons and the expression of pro-inflammatory factors (Qin and Dallas 2019).

The 1500-m freestyle is the longest pool-based swimming event in the Olympic Games. Some of the key aspects in the 1500-m freestyle performance include how to effectively improve energy utilization during competition, and how to delay the occurrence of exercise-induced fatigue. Exercise-induced fatigue is a common physiological phenomenon in which the body cannot continue to maintain a certain level or a predetermined intensity during exercise, resulting in a decline in exercise capacity (Russell et al. 2020; Kieran et al. 2018). The occurrence of exercise fatigue is often viewed as a common and complex phenomenon caused by abnormal neuromuscular functions, hormone disorders, protein imbalance, increased inflammation and oxidative stress, and overtraining (Chen et al. 2019; Arthur et al. 2017; Wu and Liu 2018). The essence of these abnormalities results from the decrease in synaptic excitability of the central nervous system (CNS) and the energy metabolism of skeletal muscles, which leads to disturbances in homeostasis and susceptibility to injury (Jian et al. 2012).However, studies on the expression of circulating EV-miRNAs after fatiguing exercise and the changes in plasma EV-miRNAs after 1500 m all-out freestyle swimming are still scarce (Lipinska et al. 2015).

EV-miRNAs may be involved in the adaptation to exercise-induced fatigue. A recent study on rats suggested that the expression of EVs carried by miR-1 increased after exercise, while the expressions of miR-133a, miR-133b, miR-206, miR-208a, and miR-499 increased immediately after, but returned to the baseline level after 48 h. The rats’ exosomes level remained unchanged at 24 h after 4 weeks of swimming training, but a significant increase was observed immediately after exercise. However, the expression of exosomes miR-1, miR-486, miR-208a, miR-3571, miR-122, miR-196b, miR-3591, miR-184, and miR-760 recovered after 24 h (Brisamar et al. 2020).These results provide evidence for physiological adaptations to physical activity in EV-miRNAs (Lovett et al. 2018; D’Souza et al. 2018). There is a relative paucity of studies specifically relating to the changes of EVs in plasma and the biological characteristics of miRNAs in EVs under an exercise-induced fatigue state. Therefore, the aim of the study was to reveal the characteristics of the EV-miRNAs in the plasma of swimmers in a state of exercise-induced fatigue, and provide a theoretical basis for finding new biomarkers of exercise fatigue. We hypothesized that the amount of EV-miRNAs in plasma would increase after a full 1500-m freestyle swimming session, and the differential profiles of circulating EV-miRNAs would be modified by the fatiguing freestyle swimming.

## Materials and methods

### Subjects

Thirteen male freestyle swimmers were recruited from Guangzhou Sport University. Inclusion criteria included: (1) nondrinker and nonsmoker; (2) non-fatigue in the past 2 days. Each subject was informed of the research procedures and objectives verbally and in writing, and a written informed consent form was signed by each participant. The study was approved by Guangzhou Sports University Ethics Committee (Approval No. 2020LCLL-006). The physical characteristics of the participants are summarized in Table 1.

**Table 1:** Subject characteristics (*N* = 13)

Age (y)	Height (cm)	Weight (kg)	BMI (kg/m^2^)	HR (bpm)	Training (y)
19.15 ± 1.07	180.31 ± 5.04	76.15 ± 9.72	24.25 ± 2.92	58.00 ± 4.76	8.54 ± 1.27

*BMI* body mass index; *HR* heart rate; *Training* years of swimming training

### Experimental program

One week before the swimming session, a baseline Wingate anaerobic power test was conducted on a MONARK-894E power bicycle (Sweden), consisting of 30 s of fastest possible pedaling at a power load of the athlete’s weight (kg) × 0.075. The maximum power (PP) and the relative maximum power (PP/kg) were recorded. Power decline (PD), relative power decline (PD/kg), power decline rate (FIpp), and fatigue percentage (PD%) were used to evaluate the anaerobic capacity of subjects.

In the morning of the swimming session, fasting venous blood samples were collected. After reporting a rating of perceived exertion (RPE) value and obtaining heart rate data with a heart rate monitor (POLAR RC3, Finland), all subjects commenced a 1500-m freestyle swimming session at the speed of their best previously recorded swimming performance (Matthews et al. 2016). After the swimming session, the RPE scale was recorded to monitor the degree of physical fatigue (Borg 1982); venous blood samples were collected again, then the second Wingate anaerobic power test was conducted.

### Sample collection and pretreatment

Within half an hour after the collection of venous blood samples, serum components were separated by centrifugation at 1000*g* for 15 min, and stored in a freezer at −80 °C before further analysis.

### Biochemical index assay

Blood lactic acid (Bla) was measured with EFK semi-automatic lactic acid analyzer (EFK, German). Creatine kinase (CK) in serum was detected using a fully automatic biochemical analyzer (Chemray-420, Rayto, China).

### ELISA

Serum samples were taken out from the −80 °C freezer, thawed on ice and centrifuged at 4 °C with 2000*g* for 5 min. The level of Serotonin (5-HT) in serum was assayed by ELISA (ABN-KA1894 Serotonin ELISA Kit) using a Multiskan Spectrum (Thermo Scientific, USA). Samples were analyzed in duplicates.

### EVs' isolation

Umibio Extracellular vesicles extraction kits (Umibio, China) were used to isolate the EVs from plasma. 3 mL of pre-chilled PBS and 1 mL of Blood Pure Exo Solution were added to the stored supernatant. The mixture was vortexed for 1 min, incubated at 4 °C for 2 h, and centrifuged at 4 °C for 60 min at 10,000*g*. The supernatant was discarded, while the pellet rich in EV particles was resuspended with 0.5 mL of PBS. After it dissolved, the resuspension was transferred to a new centrifuge tube.

### Identification of EVs with microscope

A transmission electron microscope (TEM) was used to directly observe the characteristics and morphology of the EVs for identification. After resuspending the extracted EVs with 50–100 μL of 2% paraformaldehyde, 50 μL of the EVs suspension were placed on copper mesh and allowed to stand still at room temperature for 20 min. 1% glutaraldehyde was fixed for 5 min; 4% uranyl acetate was used to negatively stain for 5 min, then the EVs pictures were photographed. The EVs were tracked using Nanosight nanoparticle tracking analysis technology (NTA) and distinguished from other particles, and finally the concentration and particle size distribution of EVs were detected.

### Western blot

The isolated EVs were lysed with RIPA lysis buffer (Umibio, China), and the protein concentration was determined through BCA method. SDS-PAGE was performed on a 10% polyacrylamide gel and transferred to a PVDF membrane. This was sealed with 5% skimmed milk powder at room temperature for 1 h, incubated overnight at 4 °C with a primary antibody (CD63, ALIX) solution, and followed by a secondary antibody to block for 1 h at room temperature. Then, the protein bands were visualized using an enhanced chemi-luminescence (ECL) reagent.

### Total RNA extraction and concentration assay

200 μL of EVs sample were placed into a RNase-Free centrifuge tube, mixed with an equal volume of 2 × denaturing solution, and placed on ice for 5 min. 200 μL of phenol:chloroform solution were added into the tube, and then vortexed for 30 s, centrifuged at 10,000*g* for 5 min at 4 °C. The supernatant was transferred to a new centrifuge tube, mixed with 1.25-fold volume of absolute ethanol, then 700 μL of the solution was transferred to the spin column and centrifuged at 10,000*g* for 30 s at 4 °C until it passed through the column. 700 μL of miRNA washing solution 1 was added, and centrifuged at 10,000*g* for 30 s at 4 °C until it passed through the spin column. 500 μL of washing solution 2 was added, and centrifuged at 10,000*g* for 30 s at 4 °C until it passed through the spin column. The supernatant was discarded and the spin column was put back into the collection tube. The resulting solution was centrifuged at 10,000*g* for 1 min at 4 °C until it all passed through the spin column. The adsorption column was put into a new collection tube and 50 μL of preheated washing solution were added at 95 °C. The precipitate (total RNA) was collected by centrifugation for 30 s, and the concentration of the total RNA extracted from EVs was detected by means of an Agilent 2100 Bioanalyzer System.

### miRNA quality detection and library construction

The 3′ and 5′ adapters were connected. 2 μL of QIAseq miRNA NGS RT Initiator were added for RNA reverse transcription, and thoroughly mixed with QIAseq beads and QIAseq miRNA NGS Bead Binding Buffer for magnetic beads (QMN Beads) preparation. cDNA synthesis and purification were performed on ice, followed by library amplification. PCR product fragments were screened, and the concentration of the library was detected using Qubit dsDNA HS assay. The Agilent 2100 Bioanalyzer High Sensitivity DNA Assay was used to detect the fragment distribution range of the library. The main peak of the library was ~ 170–180 bp. Finally, the high-throughput Illumina 2 × 150 bp platform was used to detect the total miRNA extracted from EVs (Dillies et al. 2013).

### Differential expressions of miRNAs

Using the Expdiff method, the known miRNAs in EVs was counted to determine whether there were significant differences in the expression levels between EVs, and the levels of miRNAs co-expressed between samples were compared using log_2_(fold-change) and scatter plots.

### MiRNA pathway enrichment and datum analysis

The R language package DEGseq was used to identify differentially expressed genes. the R package based on differential expression analysis of negative binomial distribution, and the analysis of differentially expressed genes with biological repetition. Three database tools: targetscan, miRanda, and PITA were used to analyze the genetic sequence information of the subjects with known miRNAs and newly predicted, differentially expressed miRNAs (Wang et al. 2021; Zhang 2021). Finally, Gene Ontology (GO) and Kyoto Encyclopedia of Genes and Genomes (KEGG) enrichment analysis were performed on the set of miRNA target genes (Ashburner et al. 2000; Minoru et al. 2004). We used E-data software to select the RT and ACC data generated by Stroop task stimulus and imported it into WPS Excel 2019 to record.

### Statistical analysis

All experimental data were recorded as mean ± standard deviation (Mean ± SD), and SPSS 23.0 software was used for statistical analysis. Each index before and after the exercise was analyzed by paired sample *T*-test, and the statistical significance was expressed at the *p* < 0.05 or *p* < 0.01 level. Graphpad Prism 7.0 software was employed for image drawing.

## Results

### HR, RPE results

The mean HR level of the subjects after a full 1500-m freestyle exercise was > 185 bpm. The RPE value was higher than 19, an extremely high level and a significant increase compared to the baseline (RPE 9).

### Bla, CK, 5-HT test results

**Fig. 1:**
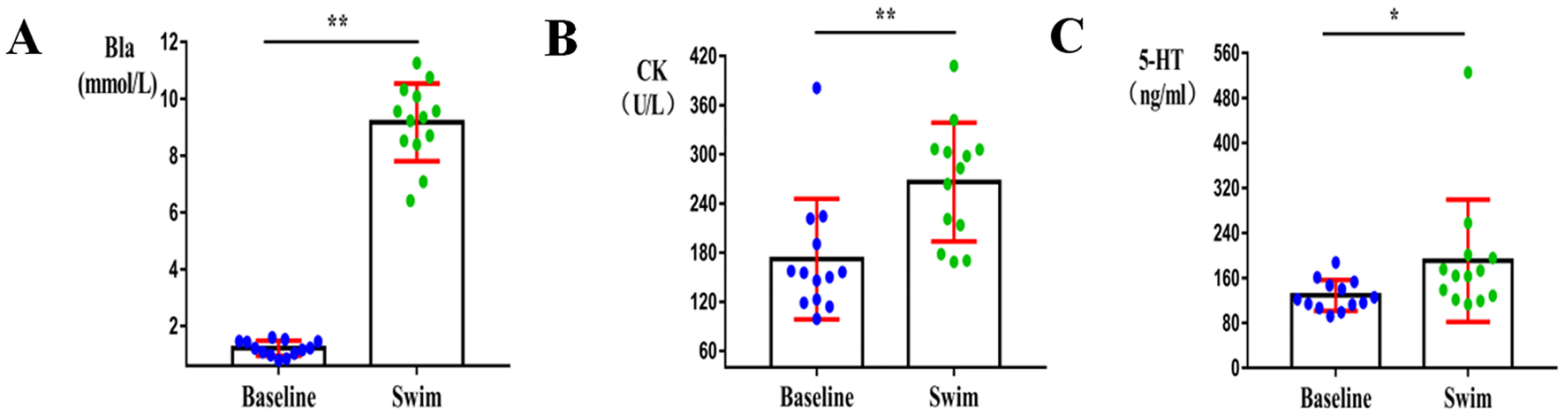
Changes in subjects' blood indicators; *p < 0.05, **p < 0.01 compared with baseline. *CK* creatine kinase;* Bla* blood lactic acid; *5-HT* serotonin. **A** Bla significantly increased, *p* < 0.01. **B** The CK of the subjects increased significantly, *p* < 0.01. **C** The levels of 5-HT significantly increased, *p* < 0.05

The subjects’ Bla significantly increased immediately after the full 1500-m freestyle exercise (*p* < 0.01) (Fig. 1A), reaching 11.2 mmol/L. The CK of the subjects increased significantly after the 1500-m exercise (*p* < 0.01) (Fig. 1B), reaching a maximum of 340 U/L.

As a central neurotransmitter in the brain, the concentration of 5-HT, one of the criteria for central nervous system fatigue, significantly increased after exercise (*p* < 0.05) (Fig. 1C).

### Anaerobic test results

**Fig. 2:**
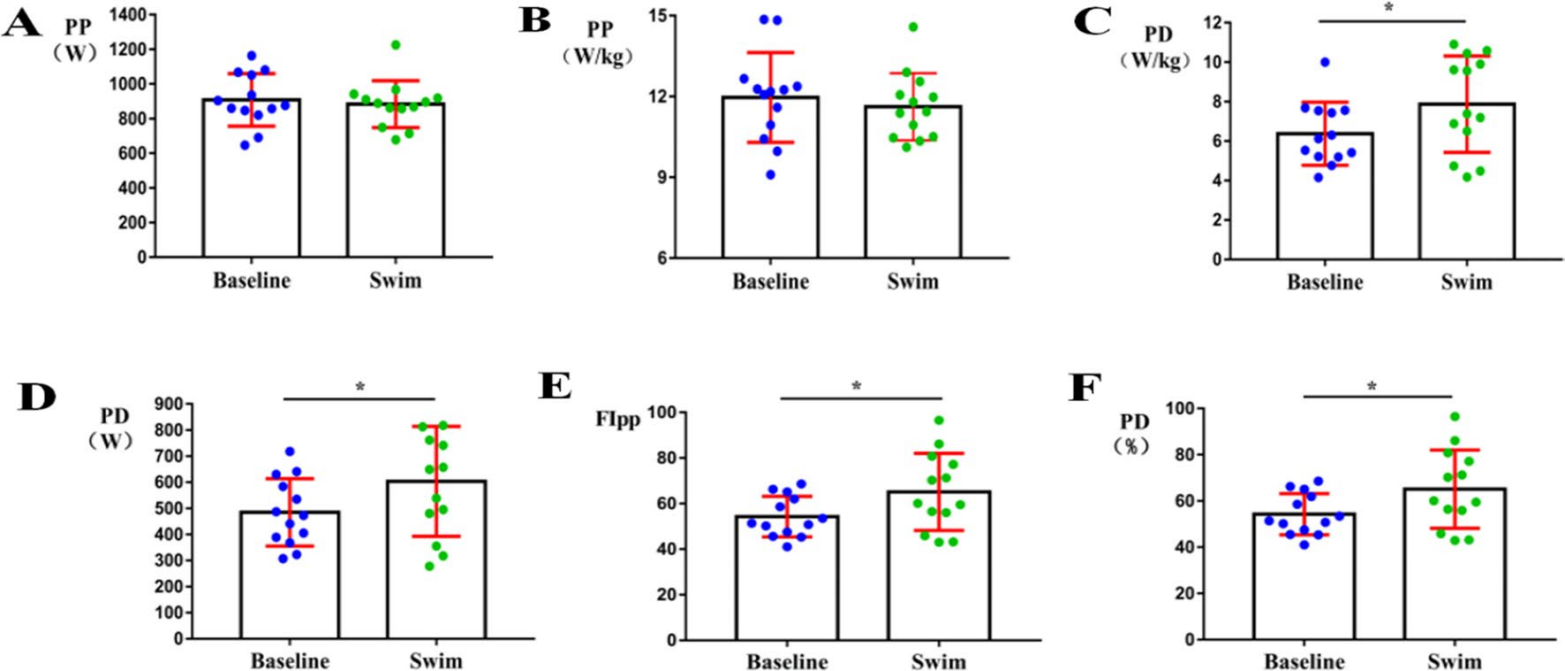
Changes in the subjects’ anaerobic capacity indicators as compared with baseline. PP (W), maximum power; PP (W/kg), relative maximum power; PD (W), power decline; PD (W/kg), relative power decline; FIpp, power decline rate; PD (%), fatigue percentage. **A** The subjects' PP (w) did not change significantly. **B** The subjects' PP (w/kg) did not change significantly. **C** The subjects' PD (w) significantly increased at *p* < 0.05. **D** The subjects' PD (w/kg) significantly increased at *p* < 0.05. **E** The subjects' FIpp significantly increased at *p* < 0.05. **F** The subjects' PD (%) significantly increased at *p* < 0.05

After the full 1500-m freestyle exercise, no significant changes were observed in the subjects' PP (w) and PP (w/kg) (Fig. 2A, B). In sharp contrast, a series of anaerobic exercise metrics significantly increased at the *p* < 0.05 level, including subjects' PD (w), PD (w/kg), FIpp, and PD (%) (Fig. 2C–F).

### Changes of plasma EV-miRNAs in 1500-m freestyle swimmers under exercise fatigue

#### Identification results of EVs

**Fig. 3:**
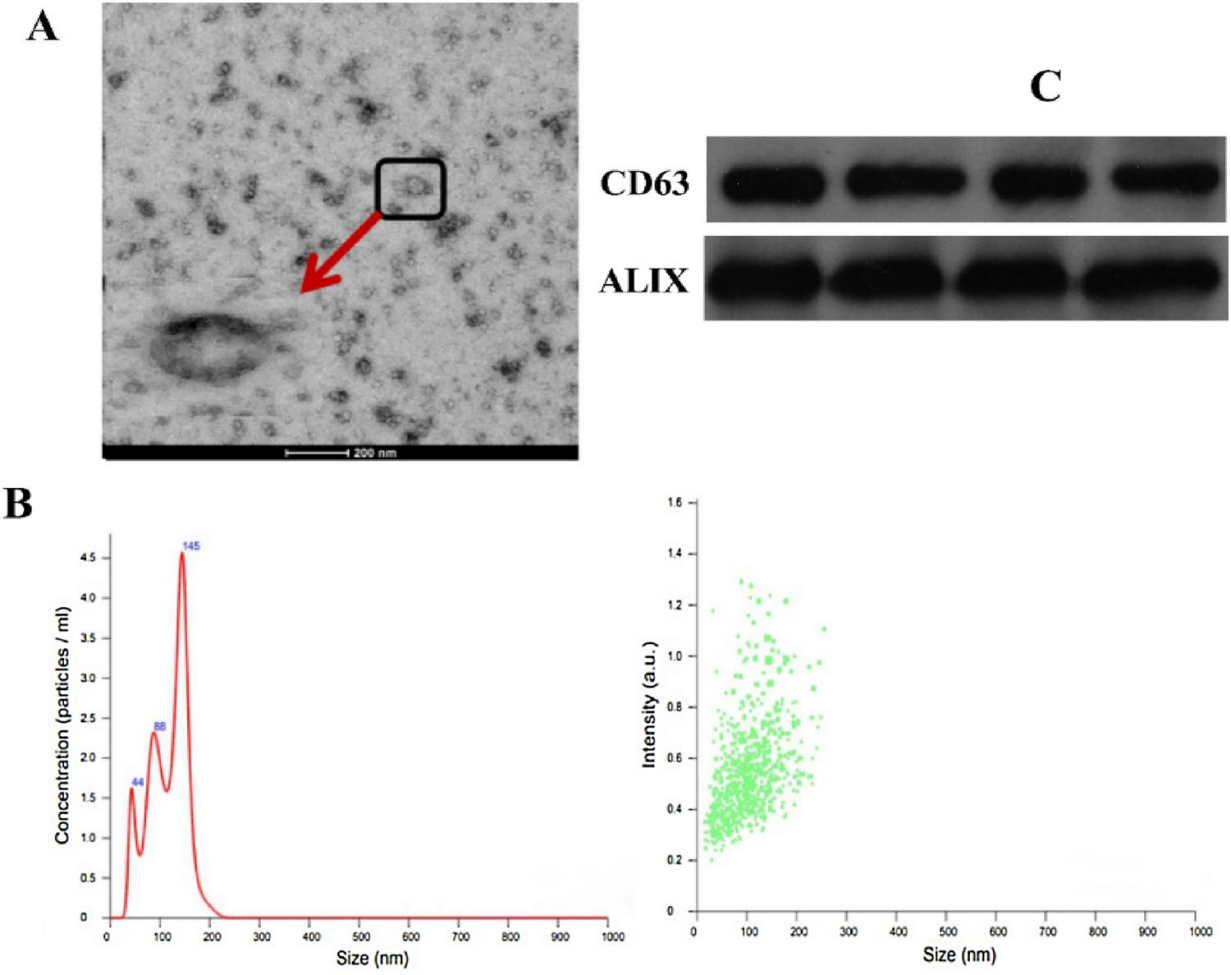
EVs in plasma identification results. **A** Image of EVs observed by TEM electron microscope. The diameter is about 200 nm. **B** NTA analysis. The abscissa is the diameter of EVs, the ordinate is their number and concentration. **C** Detection of EVs by Western blots body marker proteins CD63 and ALIX

To identify the EVs extracted from plasma samples, the morphology, particle size and distribution, and protein markers of plasma extracts were detected. Through TEM, the morphology of the extracted EVs in plasma was observed to be elliptical, and the diameter of the particles was ~ 100–200 nm (Fig. 3A). Through NTA analysis, we observed that the diameter of EV particles ranged from 40 to 200 nm, among which the particles with a diameter of 145 nm accounted for the highest proportion (Fig. 3B). The expression of EV marker proteins CD63 and ALIX were detected by WB (Fig. 3C).

#### Measurement of total RNA concentration

The concentration, total amount, and volume of total RNA were > 0.3 ng/μL, >13 ng, and 38 μL, respectively, as detected through Agilent 2100. As listed in Table 2, the quality of total miRNA extracted from the subjects' EVs in plasma met the test requirements, in that the concentration, volume, and purity of miRNAs could be satisfactorily used for database construction and subsequent trials.

**Table 2:** Total RNA concentration of EVs in plasma

Sample	Volume (μL)	Agilent 2100			Results
		Concentration (ng/μL)	Volume (μL)	Total (ng)	
I-3	98	0.347	38	13.186	Qualified
III-3	89	0.422	38	16.036	Qualified
I-6	90	0.546	38	20.748	Qualified
II-6	89	0.366	38	13.908	Qualified
I-7	89	0.546	38	20.748	Qualified
II-7	94	0.539	38	20.482	Qualified
I-11	86	0.464	38	17.632	Qualified
II-11	94	1.084	38	41.192	Qualified
I-13	92	0.352	38	13.376	Qualified
II-13	87	0.921	38	34.998	Qualified

#### Quality control of miRNAs’ detection

**Fig. 4:**
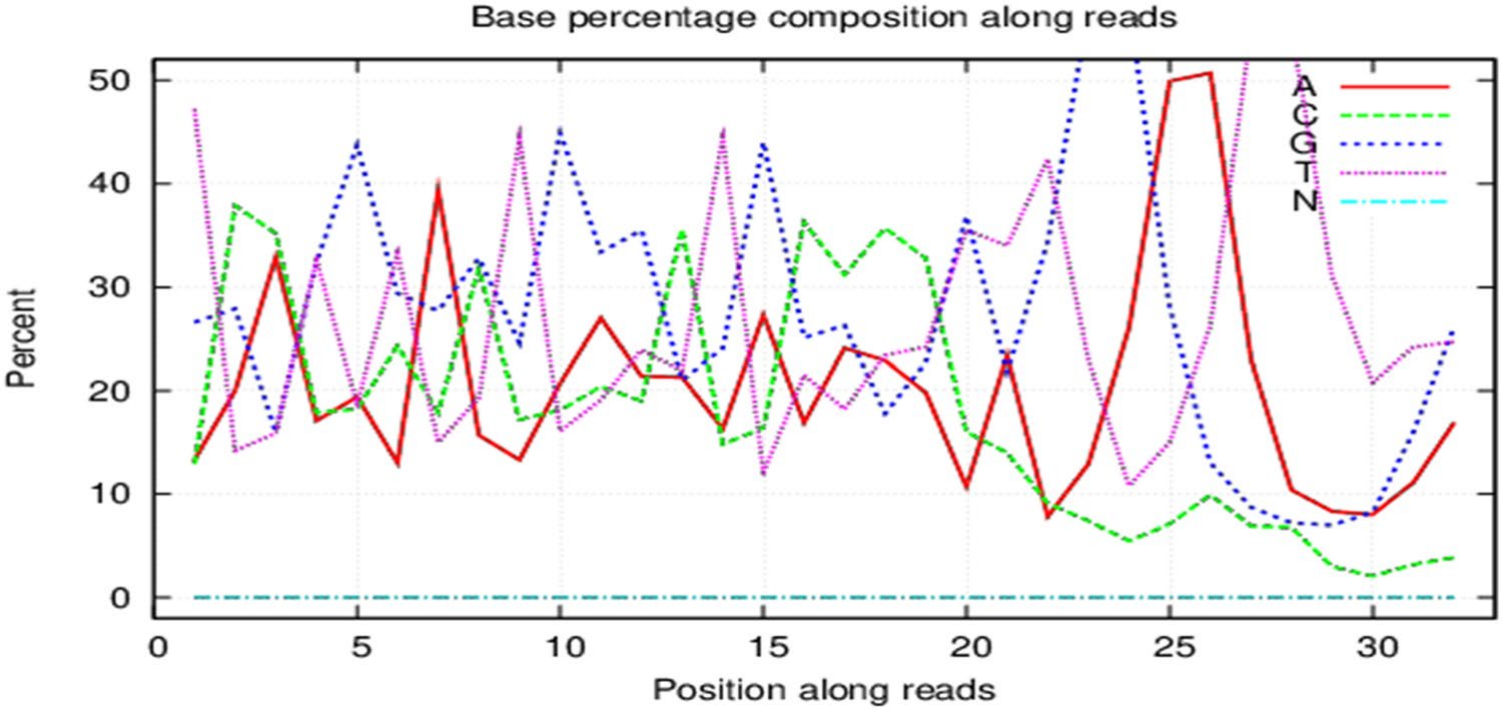
Quality control of the sample

The miRNAs’ data obtained by preliminary filtering of the original miRNAs were further filtered. The number of bases with a quality value < 20 in the filtered data exceeded 1 read, and high-quality reads were obtained. After filtering out reads containing polyA and greater than 70% of the base reads, the small RNA clean tags sequence that could be used for subsequent analysis was finally acquired (Fig. 4).

#### The differential expressions of miRNAs in EVs

By integrating high-throughput sequencing with the three miRNAs’ target gene prediction databases (PITA, Targetscan, and miRand), subjects’ plasma EV-miRNAs expressions were analyzed in detail before and after exercise. In plasma, EV-miRNAs with a value of *p* < 0.05 and │log_2_(fold-change)│> 1 were considered to be differentially expressed. In total, 70 EV-miRNAs were found with significantly differential expression, among which 45 and 25 miRNAs were up-regulated and down-regulated, respectively (see Table 3). Three miRNAs were screened out due to their up-regulated expression after exercise and fold change being > 11, which included miR-144-3p, miR-145-3p, and miR-509-5p. Two miRNAs (miR-891b and miR-890) were filtered out due to their down-regulated expression and their fold change being > 9. Subsequently, the target genes regulated by the aforementioned five miRNAs were predicted prior to functional enrichment analysis.

**Table 3:** Differentially expressed profiles of EV-miRNAs

Name	Baseline	Swim	│log_2_FC│	Up/down
hsa-miR-144-3p	0.01	48.75166	12.25124	Up
hsa-miR-145-3p	0.01	37.47716	11.8718	Up
hsa-miR-509-5p	0.01	27.3532	11.41749	Up
hsa-miR-514b-5p	0.01	18.60436	10.86143	Up
hsa-miR-382-5p	0.01	10.52996	10.04028	Up
hsa-miR-495-3p	0.01	9.47388	9.887812	Up
hsa-miR-323a-3p	0.01	8.48148	9.728172	Up
hsa-miR-486-5p	22.5453	16608.1	9.524845	Up
hsa-miR-937-3p	0.01	5.83196	9.187837	Up
hsa-miR-432-5p	0.01	4.92952	8.945303	Up
hsa-miR-139-3p	0.01	3.56202	8.476552	Up
hsa-miR-451a	75.38478	23397.58	8.27787	Up
hsa-miR-329-3p	0.01	2.52236	7.97863	Up
hsa-miR-6509-5p	0.01	2.43168	7.92581	Up
hsa-miR-5699-5p	0.01	2.2845	7.835735	Up
hsa-miR-130b-3p	0.1075	19.8096	7.525719	Up
hsa-miR-145-5p	0.59878	66.87406	6.803277	Up
hsa-miR-143-3p	22.30074	2405.706	6.753225	Up
hsa-miR-214-3p	0.45108	23.01504	5.67305	Up
hsa-miR-142-5p	16.95878	477.292	4.814768	Up
hsa-miR-199a-3p	21.09196	534.1622	4.662513	Up
hsa-miR-199b-3p	21.09196	534.1622	4.662513	Up
hsa-miR-126-3p	36.12228	900.8692	4.640357	Up
hsa-miR-3679-5p	0.3065	6.39888	4.38386	Up
hsa-miR-1304-5p	0.45108	8.2739	4.197112	Up
hsa-miR-155-5p	21.87208	381.4561	4.124355	Up
hsa-miR-127-3p	0.50548	8.30728	4.03865	Up
hsa-miR-16-5p	729.2913	8404.447	3.526586	Up
hsa-miR-146a-5p	78.67734	676.7992	3.104708	Up
hsa-miR-223-3p	103.7061	858.5918	3.049472	Up
hsa-miR-503-5p	8.49	57.74774	2.765928	Up
hsa-miR-7-5p	85.14492	426.3318	2.323984	Up
hsa-miR-574-5p	87.84664	410.3965	2.223959	Up
hsa-miR-193a-5p	120.1112	489.9138	2.028157	Up
hsa-miR-1301-3p	34.95236	128.6871	1.880406	Up
hsa-miR-21-5p	20098.81	70769.04	1.816008	Up
hsa-miR-378a-3p	228.0371	648.5802	1.508016	Up
hsa-miR-320a-3p	3761.677	10578.35	1.491667	Up
hsa-let-7i-5p	2486.93	6794.987	1.450105	Up
hsa-let-7b-5p	21096.4	57066.43	1.435645	Up
hsa-miR-181b-5p	199.5891	539.3951	1.43431	Up
hsa-miR-132-3p	52.96718	141.6074	1.418726	Up
hsa-miR-15b-5p	246.7351	598.1228	1.277479	Up
hsa-miR-128-3p	312.1267	754.5679	1.273519	Up
hsa-miR-664a-5p	72.52922	151.0079	1.05799	Up
hsa-miR-891b	8.1684	0.01	9.730463	Down
hsa-miR-890	7.97184	0.01	9.67391	Down
hsa-miR-892b	8.49496	0.01	9.638769	Down
hsa-miR-92a-2-5p	6.33392	0.01	9.306955	Down
hsa-miR-548o-3p	6.26946	0.01	9.292197	Down
hsa-miR-20a-3p	4.89706	0.01	8.935772	Down
hsa-miR-888-5p	171.1154	0.3672	8.864188	Down
hsa-miR-874-5p	4.03866	0.01	8.657733	Down
hsa-miR-3144-3p	2.3336	0.01	7.866413	Down
hsa-miR-891a-5p	464.5873	13.62056	5.092092	Down
hsa-miR-577	12.3276	0.4141	4.895769	Down
hsa-miR-892a	15.66568	0.55072	4.830145	Down
hsa-miR-497-5p	3.84982	0.1889	4.349096	Down
hsa-miR-187-3p	20.80978	1.65506	3.652306	Down
hsa-miR-582-5p	38.0094	4.47352	3.086874	Down
hsa-miR-653-3p	32.38488	4.47938	2.853949	Down
hsa-miR-590-3p	17.02644	2.61344	2.703755	Down
hsa-miR-187-5p	13.6263	2.1745	2.647638	Down
hsa-miR-628-5p	10.29026	2.69618	1.932291	Down
hsa-miR-1296-5p	12.02364	4.1779	1.525024	Down
hsa-miR-30b-5p	10245.37	3857.633	1.409185	Down
hsa-miR-1306-5p	43.10136	19.03914	1.178765	Down
hsa-miR-141-3p	412.5014	195.7189	1.075616	Down
hsa-miR-9-5p	753.6843	363.8622	1.050568	Down
hsa-miR-10a-5p	87953.51	42629.77	1.04488	Down

#### Functional enrichment analyses of target genes regulated by EV-miRNAs

The five target genes of miR-144-3p, miR-145-3p, miR-509-5p, miR-891b and miR-890 were predicted to be differentially expressed through three databases of PITA, Targetscan, and miRand. Using GO and KEGG databases the functional annotation was analyzed to find the intersection relationship.

**Fig. 5:**
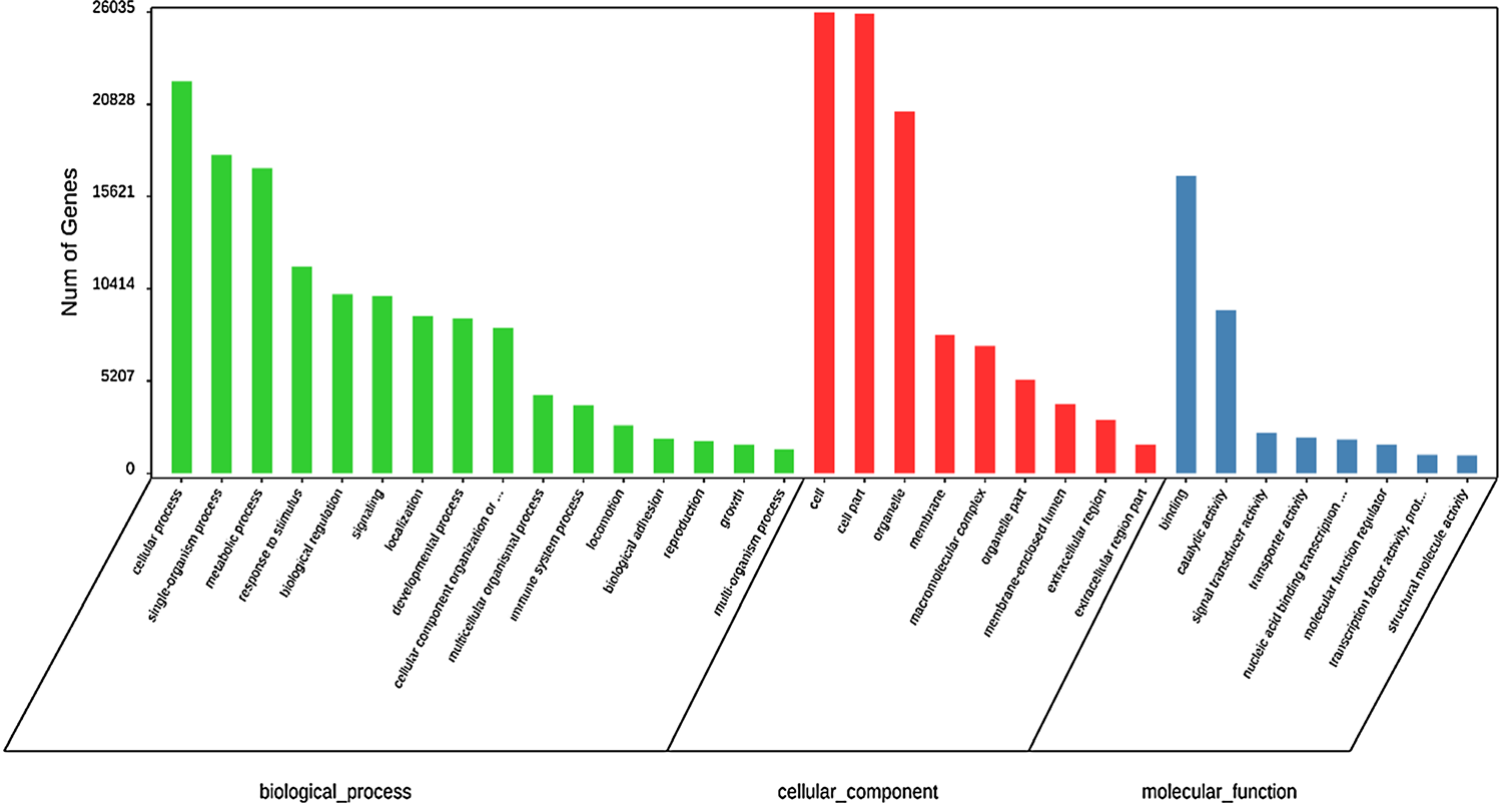
Differentially expressed EV-miRNAs GO function classification map

Figure 5 shows the statistical results of the comparison and classification of the target genes of differentially expressed EV-miRNAs in plasma through the GO database. In GO enrichment, three aspects are involved: biological process, cell composition, and molecular function, and each aspect is composed of eight items. Target genes are involved in cell metabolism, biological regulation, and signal transmission. They are mainly distributed in cell parts, membrane-enclosed cavities, and extracellular areas, and become enriched in molecular functions such as signal transmission, protein binding, and structural molecular activity.

The abscissa is the GO annotation, and the ordinate represents the number of genes. Green represents the biological process, red the cell composition, and blue the molecular function.

**Fig. 6:**
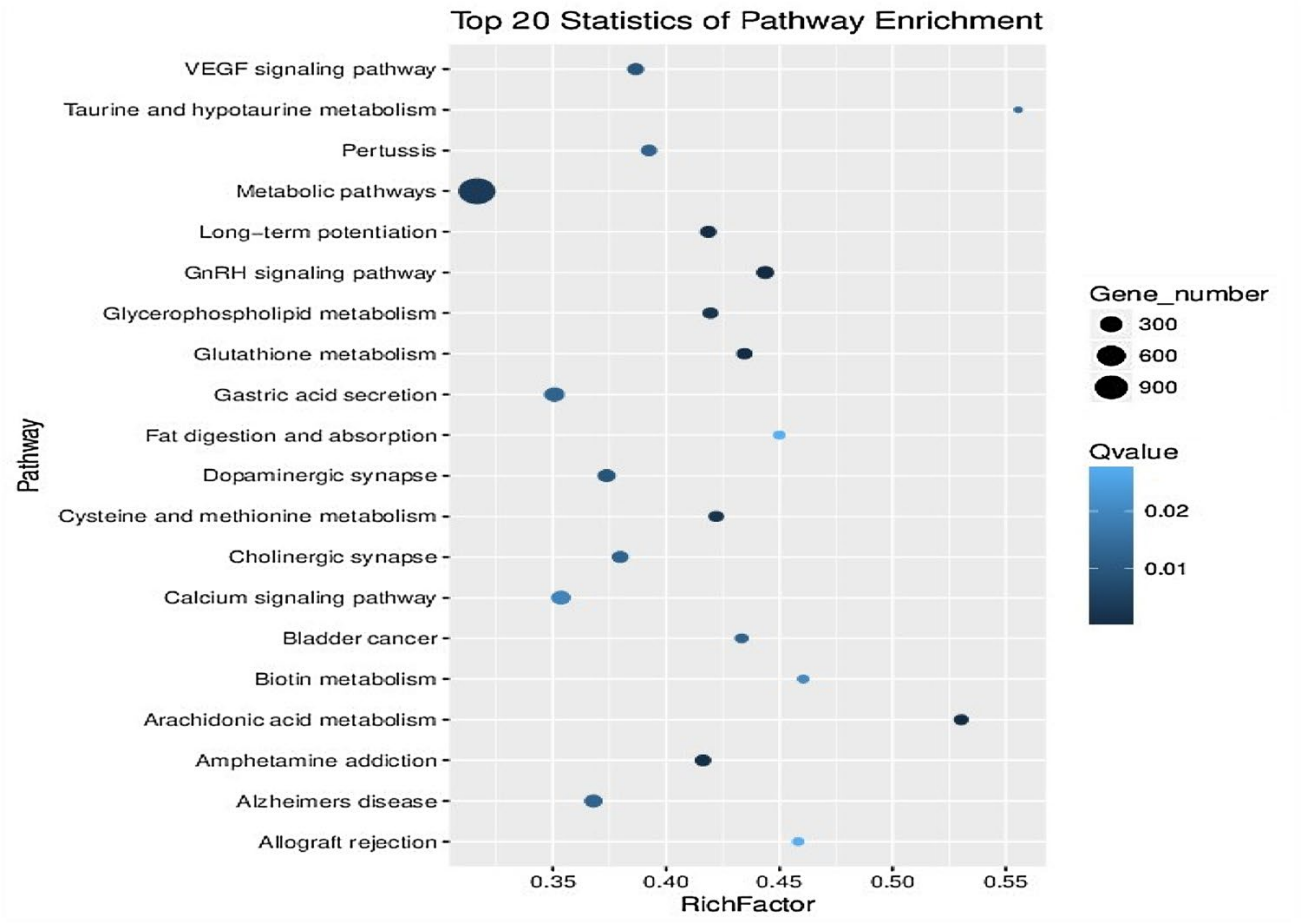
Differentially expressed EV-miRNAs target gene KEGG signaling pathway enrichment map

By screening the significantly enriched KEGG signaling pathways, the target genes regulated by the EV-miRNAs become mainly enriched in metabolic, calcium signaling, GnRH signaling and VEGF signaling pathways; long-term enhancement mechanism (long-term potentiation, or LTP); dopaminergic and cholinergic synapse; Alzheimer’s disease (AD); and glutathione, glycerophospholipid, and arachidonic acid metabolism, among others (Fig. 6). Consequently, it can be posited that EV-miRNAs are related to the enrichment of multiple signaling pathways, including those related to energy metabolism, skeletal muscle, central nervous system, immunity, and tumors. The database test shows that the metabolic pathways are the most significant and are closely related to EV-miRNAs target genes.

The color depth (*Q* value) indicates the enrichment degree of differentially expressed EV-miRNAs target genes in the signal pathway. The size of the circle (Gene number) denotes the number of genes with the differentially expressed EV-miRNAs target gene located under the signal pathway.

## Discussion

Exercise fatigue is the main factor affecting athlete's training and competition status. In this study, we used the evaluation indicators of physiology and biochemistry to comprehensively observe the fatigue of freestyle athletes after a 1500-m freestyle swimming session. The profiles of plasma EV-miRNAs under fatigue condition was obtained by high-throughput detection technology, and the differentially expressed EV-miRNAs were screened out. It was found that 70 miRNAs changed differentially after exercise, 45 miRNAs were up-regulated, and 25 miRNAs were down-regulated. By screening the five target genes of miR-144-3p, miR-145-3p, miR-509-5p, miR-891b, and miR-890 with the largest fold change, functional enrichment analysis showed that the target genes are involved in the regulation of LTP, VEGF, glutathione metabolism pathway, dopaminergic synapse, signal transmission, biological regulation, and other biological processes. Our data suggested that the miRNA profiles of circulating extracellular vesicles could be modified by a single session of fatiguing swimming, and that EV-miRNAs might be involved in the mechanisms related to adaptation to fatiguing exercise.

During the full 1500-m freestyle swimming, the serum CK value reached 340 U/L, while the blood lactate reached 11.2 mmol/L. These results show that the subjects had gone all out for the full 1500-m freestyle swimming. This study also confirmed that going all out for the full 1500-m freestyle swimming can cause exercise-induced fatigue. The RPE scale reached above 19, sports ability decreased, physical function declined, and other fatigue indicators significantly changed. Skeletal muscle is the main place for lactate production, and accumulation of lactate may be related to the calcium signal pathway regulated by EV-miRNAs, which in turn might be involved in the blockade of neuromuscular conduction (Brooks 2020).

It is interesting to note that the characteristics of EV-miRNAs were modified after a single session of fatiguing swimming. A large number of EVs were released, and the CD63 and ALIX-labeled proteins were significantly expressed in the subjects’ plasma. Similar results have been reported in the literature: the expression of EVs markers Tsg101, HSP70, CD63, and Flot-1 increased immediately following EV release after exercise on power bicycles and treadmills, and returned to baseline after 90 min of recovery (Frühbeis et al. 2015). The same phenomenon was also observed in a study of exhaustive treadmill tests (Helmig et al. 2015; Karine et al. 2018); importantly, the study also observed the expression of various miRNAs in EVs after exercise. Another study found that miR-128-3p, miR-103-3p, miR-330-5p, miR-148a-3p, miR-191a-5p, miR-10b-5p, miR-93-5p and miR-25-3p in EVs have significant differential expression after acute exercise (Oliveira Getúlio et al. 2018). In this study, it was found that five EV-miRNAs were significantly differentially expressed after fatiguing swimming; three miRNAs of miR-144-3p, miR-145-3p, miR-509-5p had been up-regulated, and two miRNAs of miR-891b and miR-890 down-regulated. Studies have shown that the changes of miRNA and proteins carried by circulating exosomes help the body cope with the stress of acute fatigue exercise (Nair et al. 2020). Differentially expressed EV-miRNAs might inhibit the post-transcriptional translation process by inhibiting mRNAs and have biological effects in the process of fatigue (Assmann et al. 2019; Görgens et al. 2015).

Of the five EV-miRNAs differentially expressed after the fatiguing swimming, MiR-144-3p is related to a variety of biological processes, including nerve function, angiogenesis, adipogenesis, bone metabolism, and tumorigenesis (Lan et al. 2015; Liu et al. 2016; Sun et al. 2017); MiR-145-3p is involved in the regulation of VEGF related to angiogenesis and LTP related to nerve function; miR-509-5p is related the GnRH signaling pathway, which is associated with the potential signal pathways of fatigue. The down-regulation of miR-891b expression can inhibit the expression of GADD45β and Lnc-IRAK3-3, which can improve the body's antiviral immunity (Liao et al. 2019). Many studies have shown that the miRNAs carried by exercise-released EVs mediate cell-to-cell communication, participate in the regulation of energy metabolism during exercise, which have been identified as novel players in promoting systemic beneficial effects (Brisamar et al. 2020; Rong et al. 2020).

Through bioinformatics analysis, it was found that the main enriched biological processes of target genes that differentially express EV-miRNAs include regulation of metabolic processes, neuromuscular signal transmission, central nervous system regulation, and biological regulation involving EVs. Through KEGG functional enrichment analysis, it was found that the target genes that differentially express EV-miRNAs are mainly involved in metabolic pathways, VEGF, LTP, dopaminergic and cholinergic synapse, calcium signaling pathway, glutathione, glycerophospholipid, and arachidonic acid metabolism, and many other pathways. The LTP signaling pathway, dopaminergic synaptic pathway, and cholinergic synapse involved in the regulation of the target genes can improve brain synaptic plasticity, improve brain cognitive and executive functions, and promote the recovery of neurological function. It can thus be suggested that the differential EV-miRNAs involve the adaptation to a single fatiguing swimming session (D’Souza et al. 2017). A recent study showed that after acute aerobic exercise, the expression level of exosomal miRNAs (miR206, miR133b, and miR-181a-5p) increased (Oliveira Getúlio et al. 2018). Bioinformatics pathway analysis shows that exercise-induced exosomes are predicted to target genes involved in the MAPK pathway to promote muscle cell growth and differentiation. It can be seen that the up-regulation of selective muscle-specific miRNAs in exosomes may be related to the degree of muscle damage, thereby promoting the process of muscle repair and regeneration (Muroya et al. 2015). Therefore, after fatiguing swimming, the characteristics of EV-miRNAs had changed to adapt to exercise-induced physiological changes (Safdar and Tarnopolsky 2018). Differentially expressed miRNAs might cross-regulate multiple biological information processes and signaling pathways, indicating that exercise-induced EV-miRNAs changes might play an important role in skeletal muscle regulation and central nervous system regulation, which participate in the process of fatigue.

## Conclusions

The miRNA profile of EVs in plasma changed significantly under the fatigue state after 1500-m freestyle swimming, especially for miR-144-3p, miR-145-3p, miR-509-5p, miR-891b, and miR-890. The changed EV-miRNAs might be involved in the mechanisms related to adaptation to fatiguing exercise, and can provide theoretical support for targeted prevention of exercise-induced fatigue.

### Limitations

A number of limitations of this study should be considered. The study design lacked appropriate rest and non-fatiguing exercise controls, so the present findings cannot be solely ascribed to the impact of the fatiguing 1500 m freestyle swimming. In addition, only male athletes were included in this study.

## Data Availability

The datasets for this study are available as [raw data20220606.zip] at https://figshare.com/articles/dataset/raw_data20220606_zip/20029349.
